# Supplementary material for: Laboratory surrogate markers of residual HIV replication among distinct groups of individuals under antiretroviral therapy
Source: PLoS One. 2019 Jun 17;14(6):e0217502. doi: 10.1371/journal.pone.0217502 (PMC6576780; doi:10.1371/journal.pone.0217502)
Supplement: S1 Table — The number in brackets indicates the number of patients in each scheme. (DOCX) [file pone.0217502.s001.docx]

**S1 Table**

| **Group** | **1^st^ Treatment NNRTI** | **1^st^ Treatment PI/r** | **PI-r Salvage Therapy** | **PI-r and RAL Salvage Therapy** | **Virologic Failure** |
| --- | --- | --- | --- | --- | --- |
| **Treatment** | TDF- 3TC- EFV (7) | AZT- 3TC- ATV/r (11) | AZT- 3TC- TDF- LPV/r (4) | AZT- 3TC- TDF- DRV/r- RAL (1) | 3TC-TDF-LPV/r (2) |
|  | AZT- 3TC- NVP (1) | AZT- 3TC- LPV/r (6) | AZT- 3TC-TDF- ATV/r (3) | 3TC- EFV- DRV/r- RAL (1) | 3TC- TDF- DRV/r (1) |
|  | AZT- 3TC- EFV (14) | d4T-3TC-LPV/r (1) | AZT - 3TC- TDF- DRV/r (1) | 3TC- TDF- DRV/r- RAL (9) | 3TC-TDF-ATV/r (3) |
|  | d4T- 3TC- EFZ (2) | AZT-3TC-TDF-LPV/r (2) | d4T- TDF- LPV/r (1) | 3TC- TDF- DRV/r- RAL -VCV (1) | AZT- 3TC- TDF- LPV/r (1) |
|  | ddI- 3TC- EFV (1) | TDF-3TC-LPV/r (2) | AZT-3TC-ddI-DRV/r (1) | 3TC-TDF-EFV-DRV/r-RAL (1) | ABC- 3TC- LPV/r (2) |
|  | ABC-3TC-EFV (1) | TDF- 3TC- ATV/r (3) | 3TC-ABC-ATV/r (1) | 3TC- TDF- LPV/r- RAL (1) | AZT – 3TC – LPV/r (1) |
|  |  |  | d4T-ddI-LPV/r (1) | ddI- ABC- TPV/r- RAL (1) | AZT- 3TC- TDF- ATV/r (3) |
|  |  |  | TDF – 3TC – ATV/r (2) |  | 3TC-TDF-fAPV/r (1) |
|  |  |  | TDF- 3TC- DRV/r (2) |  | TDF- LPV/r (1) |
|  |  |  | TDF-3TC- ABC- ATV/r (1) |  |  |
|  |  |  | TDF-3TC-EFV- DRV/r (1) |  |  |
|  |  |  | TDF-3TC-EFV- FPV/r (1) |  |  |
|  |  |  | AZT -3TC -ATV/r (3) |  |  |
|  |  |  | AZT - 3TC -LPV/r (3) |  |  |
|  |  |  | 3TC - TDF - EFV - LPV/r (1) |  |  |
|  |  |  | 3TC - TDF - LPV/r (1) |  |  |

TDF= tenofovir; d4T= stavudine; ddI= didanosine; 3TC= lamivudine; EFV= efavirenz; AZT=zidovudine ; NVP= nelfinavir; ABC= abacavir ; ATV= atazanavir; LPV= lopinavir; DRV= darunavir; RAL= RALtegravir; r=ritonavir
