# Supplementary material for: Laboratory surrogate markers of residual HIV replication among distinct groups of individuals under antiretroviral therapy
Source: PLoS One. 2019 Jun 17;14(6):e0217502. doi: 10.1371/journal.pone.0217502 (PMC6576780; doi:10.1371/journal.pone.0217502)
Supplement: S2 Table — (DOCX) [file pone.0217502.s002.docx]

S2 Table

|  | **N** | **Gender** | **Age (years)** | | | | **CD4+ T-cell count (cells/mm^3^)** | | | | **CD8+ T-cell count (cells/mm^3^)** | | | |
| --- | --- | --- | --- | --- | --- | --- | --- | --- | --- | --- | --- | --- | --- | --- |
|  |  | Male | Mean | Maximum | Minimum | Median | Mean | Maximum | Minimum | Median | Mean | Maximum | Minimum | Median |
| **1^st^ Treatment NNRTI** | 26 | 21 | 47.17 | 63.40 | 32.20 | 50.55 | 791.3 | 1705.0 | 183.0 | 704.0 | 1033 | 1723 | 465 | 938 |
| **1^st^ Treatment PI-r** | 25 | 20 | 48.20 | 64.60 | 32.00 | 49.10 | 609.4 | 1062.0 | 330.0 | 549.0 | 990 | 2738 | 476 | 882 |
| **PI-r Salvage Therapy** | 27 | 17 | 50.01 | 82.60 | 35.90 | 46.60 | 623.3 | 1592.0 | 67.0 | 560.0 | 924 | 1941 | 300 | 782 |
| **PI-r and RAL Salvage Therapy** | 22 | 18 | 51.69 | 70.80 | 33.12 | 49.68 | 485.5 | 1308.0 | 34.0 | 420.0 | 996 | 1757 | 413 | 884 |
| **Virologic Failure** | 16 | 9 | 40.35 | 67.00 | 19.60 | 38.65 | 185.3 | 566.0 | 1.0 | 126.0 | 828 | 2444 | 51 | 720 |
| **Total** | 116 | 85 | 47.97 | 82.60 | 19.60 | 48.33 | 571.4 | 1705.0 | 1.0 | 519.0 | 963 | 2738 | 51 | 873 |

NNRTI= non-nucleoside analog reverse-transcriptase inhibitor, PI-r = ritonavir boosted protease inhibitor, and RAL = Raltegravir.
