# Supplementary material for: Laboratory surrogate markers of residual HIV replication among distinct groups of individuals under antiretroviral therapy
Source: PLoS One. 2019 Jun 17;14(6):e0217502. doi: 10.1371/journal.pone.0217502 (PMC6576780; doi:10.1371/journal.pone.0217502)
Supplement: S3 Table — (DOCX) [file pone.0217502.s003.docx]

S3 Table

|  | **Time (years) with undetectable viral load** | | | | **Viral load (log_10_ HIV copies/mL)** | | | | **Number of treatment schemes** | | | | **Number of drugs** | | | |
| --- | --- | --- | --- | --- | --- | --- | --- | --- | --- | --- | --- | --- | --- | --- | --- | --- |
|  | Mean | Maximum | Minimum | Median | Mean | Maximum | Minimum | Median | Mean | Minimum | Maximum | Median | Mean | Minimum | Maximum | Median |
| **1^st^ Treatment NNRTI** | 6.43 | 14.60 | 2.30 | 5.60 | - | - | - | - | - | - | - | - | - | - | - | - |
| **1^st^ Treatment PI-r** | 7.14 | 12.70 | 1.00 | 6.60 | - | - | - | - | - | - | - | - | - | - | - | - |
| **PI-r Salvage Therapy** | 6.81 | 13.60 | 1.00 | 7.60 | - | - | - | - | 6 | 1 | 11 | 6 | 9 | 4 | 14 | 10 |
| **PI-r and RAL Salvage Therapy** | 3.70 | 7.50 | 1.10 | 3.60 | - | - | - | - | 8 | 1 | 13 | 7 | 12 | 5 | 19 | 13 |
| **Virologic Failure** | - | - | - | - | 4.66 | 5.45 | 2.16 | 3.68 | 5 | 2 | 14 | 4 | 9 | 6 | 15 | 7 |
| **Total** | 6.13 | 14.60 | 1.00 | 5.60 | 4.66 | 5.45 | 2.16 | 3.68 | - | - | - | - | - | - | - | - |

NNRTI= non-nucleoside analog reverse-transcriptase inhibitor, PI-r = ritonavir boosted protease inhibitor, and RAL = Raltegravir
