# Supplementary material for: Laboratory surrogate markers of residual HIV replication among distinct groups of individuals under antiretroviral therapy
Source: PLoS One. 2019 Jun 17;14(6):e0217502. doi: 10.1371/journal.pone.0217502 (PMC6576780; doi:10.1371/journal.pone.0217502)
Supplement: S4 Table — (DOCX) [file pone.0217502.s004.docx]

S4 Table

| **Sample ID** | **Number of PBMCs extracted** | **Total HIV DNA (copies /10^6^ cells)** | **Number of cells examined for Total HIV DNA** | **2-LTR (copies /10^6^ cells)** | **Number of cells examined for 2-LTR** |
| --- | --- | --- | --- | --- | --- |
| 1 | 3,623,372.00 | ND | 36,233.72 | 22.15 | 579,739.52 |
| 2 | 4,791,164.00 | 159.48 | 47,911.64 | 28.84 | 766,586.24 |
| 3 | 28,328,228.00 | 2.95 | 283,282.28 | ND | 4,532,516.48 |
| 4 | 4,765,316.00 | ND | 47,653.16 | 191.44 | 762,450.56 |
| 5 | 5,281,302.00 | 194.23 | 52,813.02 | ND | 845,008.32 |
| 6 | 17,770,556.00 | 169.85 | 177,705.56 | ND | 2,843,288.96 |
| 7 | 42,833,388.00 | 149.1 | 428,333.88 | 0.42 | 6,853,342.08 |
| 8 | 39,480,722.00 | 89.77 | 394,807.22 | ND | 6,316,915.52 |
| 9 | 43,923,066.00 | 205.34 | 439,230.66 | ND | 7,027,690.56 |
| 10 | 15,502,340.00 | 77.34 | 155,023.40 | 17.12 | 2,480,374.40 |
| 11 | 23,532,600.00 | 107.9 | 235,326.00 | ND | 3,765,216.00 |
| 12 | 29,039,879.40 | 16.02 | 290,398.79 | ND | 4,646,380.70 |
| 13 | 17,720,834.60 | 106.87 | 177,208.35 | 10.17 | 2,835,333.54 |
| 14 | 30,152,669.40 | 69.35 | 301,526.69 | ND | 4,824,427.10 |
| 15 | 25,268,075.00 | 109.58 | 252,680.75 | 12.19 | 4,042,892.00 |
| 16 | 20,183,961.84 | 78.92 | 201,839.62 | 21.05 | 3,229,433.89 |
| 17 | 35,896,070.00 | 122.72 | 358,960.70 | 0.78 | 5,743,371.20 |
| 18 | 4,768,629.60 | 372.93 | 47,686.30 | ND | 762,980.74 |
| 19 | 20,573,043.82 | 156.83 | 205,730.44 | ND | 3,291,687.01 |
| 20 | 14,906,760.26 | 72.33 | 149,067.60 | ND | 2,385,081.64 |
| 21 | 14,144,331.32 | 26.76 | 141,443.31 | ND | 2,263,093.01 |
| 22 | 20,665,898.82 | 34.75 | 206,658.99 | 1.95 | 3,306,543.81 |
| 23 | 16,451,447.04 | 164.22 | 164,514.47 | ND | 2,632,231.53 |
| 24 | 12,340,623.82 | 74.25 | 123,406.24 | ND | 1,974,499.81 |
| 25 | 21,265,989.08 | ND | 212,659.89 | ND | 3,402,558.25 |
| 27 | 23,608,309.34 | 101.15 | 337,261.56 | 2.1 | 4,531,345.36 |
| 28 | 22,803,469.08 | 68.06 | 325,763.84 | 1.48 | 4,376,865.47 |
| 29 | 22,408,623.16 | 148.11 | 320,123.19 | ND | 4,301,079.30 |
| 30 | 21,298,156.32 | 52.92 | 304,259.38 | 0.88 | 4,087,937.87 |
| 31 | 17,288,053.16 | 186.58 | 246,972.19 | ND | 2,766,088.51 |
| 32 | 22,535,804.34 | 1021.69 | 321,940.06 | ND | 3,605,728.69 |
| 33 | 21,945,002.24 | 71.8 | 313,500.03 | 6.94 | 3,511,200.36 |
| 34 | 26,114,900.00 | 84.66 | 373,070.00 | ND | 4,178,384.00 |
| 35 | 19,110,881.06 | 272.34 | 216,676.66 | 1.44 | 3,239,132.38 |
| 36 | 18,647,773.20 | 98.03 | 211,426.00 | 12.91 | 3,160,639.53 |
| 37 | 23,110,995.02 | 42.72 | 262,029.42 | ND | 3,917,117.80 |
| 38 | 26,363,211.61 | 24.66 | 298,902.63 | ND | 4,468,340.95 |
| 39 | 30,274,955.88 | 20.41 | 343,253.47 | ND | 5,131,348.45 |
| 40 | 27,949,827.48 | 29.06 | 316,891.47 | ND | 4,737,258.89 |
| 41 | 16,124,332.00 | 69.59 | 230,347.60 | ND | 2,579,893.12 |
| 42 | 25,023,026.00 | 16.55 | 357,471.80 | ND | 4,003,684.16 |
| 43 | 26,277,174.00 | 25.23 | 375,388.20 | ND | 4,204,347.84 |
| 44 | 17,749,956.00 | 65.31 | 253,570.80 | ND | 2,839,992.96 |
| 45 | 23,685,046.00 | 68.6 | 338,357.80 | 5.99 | 3,789,607.36 |
| 46 | 23,781,114.00 | 87.9 | 339,730.20 | ND | 3,804,978.24 |
| 47 | 16,102,366.00 | 140.45 | 230,033.80 | ND | 2,576,378.56 |
| 48 | 18,296,040.00 | 44.9 | 261,372.00 | ND | 2,927,366.40 |
| 49 | 24,223,220.00 | 147.36 | 346,046.00 | 5.13 | 3,875,715.20 |
| 50 | 22,465,436.00 | 127.81 | 320,934.80 | ND | 3,594,469.76 |
| 51 | 11,130,309.40 | 1017.46 | 159,004.42 | ND | 1,780,849.50 |
| 52 | 11,781,632.80 | 162.96 | 168,309.04 | 106.86 | 1,885,061.25 |
| 53 | 18,996,534.40 | 23.04 | 379,930.69 | ND | 3,039,445.50 |
| 54 | 17,640,098.00 | 73.49 | 252,001.40 | ND | 2,822,415.68 |
| 55 | 19,142,508.00 | 23.96 | 273,464.40 | 1.79 | 3,062,801.28 |
| 56 | 26,139,484.00 | 980.73 | 373,421.20 | 5.61 | 4,182,317.44 |
| 57 | 19,845,842.24 | 123.23 | 283,512.03 | ND | 3,175,334.76 |
| 59 | 23,220,238.16 | 97.25 | 331,717.69 | ND | 3,715,238.11 |
| 60 | 18,275,928.16 | 112.46 | 261,084.69 | ND | 2,924,148.51 |
| 61 | 15,393,680.26 | 129.84 | 219,909.72 | ND | 2,462,988.84 |
| 62 | 19,335,640.66 | 123.53 | 276,223.44 | ND | 3,093,702.51 |
| 63 | 16,344,865.46 | 101.78 | 233,498.08 | ND | 2,615,178.47 |
| 64 | 26,025,440.00 | 77.63 | 371,792.00 | ND | 4,164,070.40 |
| 66 | 26,192,464.34 | 911.77 | 374,178.06 | ND | 4,190,794.29 |
| 67 | 23,320,790.92 | 117.62 | 333,154.16 | ND | 3,731,326.55 |
| 68 | 26,207,339.34 | 31.4 | 374,390.56 | ND | 4,193,174.29 |
| 69 | 24,883,998.16 | 149.89 | 355,485.69 | ND | 3,981,439.71 |
| 70 | 17,243,119.74 | 33.89 | 246,330.28 | ND | 2,758,899.16 |
| 71 | 18,579,130.92 | 104.44 | 265,416.16 | 11.7 | 3,715,826.18 |
| 72 | 18,372,534.74 | 162.64 | 262,464.78 | 7.15 | 3,674,506.95 |
| 73 | 18,915,194.34 | 70.17 | 270,217.06 | ND | 3,783,038.87 |
| 74 | 19,474,837.76 | 168.71 | 278,211.97 | ND | 3,894,967.55 |
| 75 | 16,991,189.74 | 108.94 | 242,731.28 | ND | 3,398,237.95 |
| 76 | 29,531,641.58 | 69.85 | 421,880.59 | 0.43 | 5,906,328.32 |
| 78 | 20,659,607.50 | 8.87 | 295,137.25 | ND | 4,131,921.50 |
| 79 | 25,826,788.82 | ND | 368,954.13 | 4.64 | 5,165,357.76 |
| 80 | 23,387,319.34 | 84.32 | 334,104.56 | ND | 4,677,463.87 |
| 81 | 17,681,231.12 | 62.05 | 252,589.02 | ND | 3,536,246.22 |
| 82 | 24,606,938.16 | 196.15 | 351,527.69 | ND | 4,921,387.63 |
| 83 | 15,068,006.38 | 591.52 | 215,257.23 | ND | 3,013,601.28 |
| 84 | 18,306,078.42 | 125.7 | 261,515.41 | ND | 3,661,215.68 |
| 85 | 19,507,635.00 | 95.12 | 278,680.50 | ND | 3,901,527.00 |
| 86 | 23,971,469.34 | 46.85 | 342,449.56 | ND | 3,835,435.09 |
| 87 | 14,682,157.70 | 296.31 | 209,745.11 | ND | 2,349,145.23 |
| 88 | 20,197,843.82 | 498.2 | 288,540.63 | 9.82 | 3,231,655.01 |
| 89 | 22,239,293.16 | 95.31 | 317,704.19 | ND | 3,558,286.91 |
| 90 | 29,374,688.42 | 280.06 | 419,638.41 | 6.93 | 4,699,950.15 |
| 91 | 18,207,712.04 | 95.79 | 260,110.17 | ND | 2,913,233.93 |
| 92 | 18,129,617.24 | 51.78 | 258,994.53 | ND | 2,900,738.76 |
| 93 | 15,246,276.78 | 131.07 | 217,803.95 | ND | 2,439,404.28 |
| 94 | 25,563,496.84 | 216.73 | 365,192.81 | ND | 4,090,159.49 |
| 96 | 15,670,539.08 | 128.82 | 223,864.84 | ND | 2,507,286.25 |
| 98 | 15,223,633.32 | 27.43 | 217,480.48 | ND | 2,435,781.33 |
| 99 | 16,893,539.74 | 13.21 | 241,336.28 | ND | 2,702,966.36 |
| 100 | 20,544,632.50 | ND | 293,494.75 | ND | 3,287,141.20 |
| 101 | 14,553,810.46 | 61.31 | 207,911.58 | ND | 2,328,609.67 |
| 102 | 14,921,101.58 | 52.53 | 213,158.59 | ND | 2,387,376.25 |
| 103 | 23,034,849.74 | 238.29 | 329,069.28 | 13.75 | 3,685,575.96 |
| 104 | 6,183,930.20 | 238.46 | 88,341.86 | ND | 989,428.83 |
| 105 | 13,643,437.50 | 53.18 | 194,906.25 | ND | 2,182,950.00 |
| 106 | 17,269,048.16 | 10.52 | 246,700.69 | ND | 2,763,047.71 |
| 107 | 11,895,526.58 | 197.09 | 169,936.09 | ND | 1,903,284.25 |
| 108 | 6,395,894.54 | 171.35 | 91,369.92 | ND | 1,023,343.13 |
| 109 | 17,339,026.32 | 17.71 | 247,700.38 | ND | 2,774,244.21 |
| 111 | 14,501,233.88 | 205.54 | 207,160.48 | 11.31 | 2,320,197.42 |
| 112 | 14,608,291.32 | 45.38 | 208,689.88 | 7.89 | 2,337,326.61 |
| 113 | 15,186,755.94 | 395.68 | 216,953.66 | 13.78 | 2,429,880.95 |
| 114 | 20,841,097.81 | 624.44 | 297,729.97 | 14.29 | 3,334,575.65 |
| 115 | 14,750,420.78 | 1301.25 | 210,720.30 | 13.58 | 2,360,067.33 |
| 116 | 11,444,165.60 | 123.37 | 228,883.31 | 12.71 | 1,831,066.50 |
| 118 | 5,316,417.40 | ND | 75,948.82 | 2.93 | 2,126,566.96 |
| 119 | 3,367,987.00 | 282.97 | 48,114.10 | 2.14 | 1,347,194.80 |
| 120 | 5,666,206.00 | 237.86 | 80,945.80 | 7.24 | 2,266,482.40 |
| 121 | 8,990,825.20 | 42.81 | 128,440.36 | ND | 3,596,330.08 |
| 122 | 8,075,173.40 | 135.08 | 115,359.62 | 9.36 | 3,230,069.36 |
| 123 | 9,934,671.60 | 69.78 | 141,923.88 | ND | 3,973,868.64 |
| 124 | 5,130,192.20 | 142.04 | 73,288.46 | 4.91 | 2,052,076.88 |

ND: not detected
